# Tob negatively regulates NF-κB activation in breast cancer through its association with the TNF receptor complex

**DOI:** 10.1038/s41417-025-00897-6

**Published:** 2025-04-01

**Authors:** Miho Tokumasu, Atsuko Sato, Taku Ito-Kureha, Mizuki Yamamoto, Nao Ohmine, Kentaro Semba, Jun-ichiro Inoue, Tadashi Yamamoto

**Affiliations:** 1 Cell Signal Unit, Okinawa Institute of Science and Technology Graduate University, Okinawa, Japan; 2 Department of Immunology, Okayama University Graduate School of Medicine, Dentistry, and Pharmaceutical Sciences, Okayama, Japan; 3 Department of Immunology, Graduate School of Medicine and Faculty of Medicine, The University of Tokyo, Tokyo, Japan; 4 Research Center for Asian Infectious Diseases, The Institute of Medical Science, The University of Tokyo, Tokyo, Japan; 5 Department of Life Science and Medical Bioscience, Waseda University, Tokyo, Japan; 6 The University of Tokyo Pandemic Preparedness, Infection and Advanced Research Center (UTOPIA), Tokyo, Japan

**Keywords:** Cell biology, Breast cancer

## Abstract

NF-κB mediates transcriptional regulation crucial to many biological functions, and elevated NF-κB activity leads to autoimmune and inflammatory diseases, as well as cancer. Since highly aggressive breast cancers have few therapeutic molecular targets, clarification of key molecular mechanisms of NF-κB signaling would facilitate the development of more effective therapy. In this report, we show that Tob, a member of the Tob/BTG family of antiproliferative proteins, acts as a negative regulator of the NF-κB signal in breast cancer. Studies with 35 human breast cancer cell lines reveal that Tob expression is negatively correlated with NF-κB activity. Analysis of The Cancer Genome Atlas (TCGA) database of clinical samples reveals an inverse correlation between Tob expression and NF-κB activity. Tob knockdown in human breast cancer cells promoted overactivation of NF-κB upon TNF-α treatment, whereas overexpression of Tob inhibited TNF-α stimulation-dependent NF-κB activation. Mechanistically, Tob associates with the TNF receptor complex I and consequently inhibits RIPK1 polyubiquitylation, leading to possible prevention of overwhelming activation of NF-κB.

## Introduction

Dysregulated nuclear factor-kappa B (NF-κB) is involved in malignant transformation and in maintaining the malignancy or survival of breast cancer [1, 2]. Breast cancer is classified into five subtypes [3–5]. The subtype correlates well with NF-κB activity. The luminal-like subtype, luminal A- and luminal B-like, has relatively low NF-κB activity, but aggressive subtypes, basal-like and claudin-low, generally have high NF-κB activity [1, 6]. Excessive NF-κB activity in breast cancer has been implicated in tumorigenesis and endocrine therapy resistance [2]. NF-κB activation occurs via canonical and non-canonical NF-κB signaling pathways. The canonical pathway responds immediately to environmental stimuli, leading to rapid but transient NF-κB activation, resulting in the induction of cytokines, cell proliferation, and survival. In the tumor microenvironment of the solid tumor, tumor-infiltrating lymphocytes kill cancer cells by producing cytokines, such as, interferon-gamma (IFN-γ), granzyme B, and tumor-necrosis factor-alpha (TNF-α). TNF-α is one of the most representative cytokines to induce canonical NF-κB signaling. Generally, TNF-α induces the inflammation, but the concentrated TNF-α can kill the cancer cells in vivo in the glioblastoma [7]. Therefore, the role of NF-κB and TNF-α is complex in tumor. Understanding the molecular mechanisms of NF-κB activation in breast cancer is crucial for elucidating the NF-κB targeted therapy and overcoming the therapy resistance.

Polyubiquitylation of Receptor Interacting Protein kinase 1(RIPK1) is critically important for the NF-κB canonical pathway activation [8]. When cells are stimulated by TNF-α, the TNF receptor (TNFR) bound to TNF-α moves to the lipid rafts in the plasma membrane and recruits the TNFR-associated death domain, RIPK1, cellular inhibitors of apoptosis proteins 1 and 2, and TNF-associated factor 2 to form a TNFR complex I [8, 9]. RIPK1 is immediately polyubiquitylated through M1- or Lys 63-linked ubiquitin [8, 10, 11]. The transforming growth factor-β-activated kinase 1 (TAK1) /TAK-binding protein complex is assembled at the polyubiquitylated RIPK1 scaffold. Simultaneously, NF-κB essential modulator (NEMO) is recruited to this scaffold with IκB kinase (IKK)-α/β, which is then phosphorylated by TAK1 [12–14]. The phosphorylated IKK complex causes IκB-α phosphorylation and degradation by the ubiquitin-proteasome system, resulting in the release of transcriptional factor NF-κB p65 and enhancement of target gene transcription. To subdue NF-κB activation, cylindromatosis (CYLD), A20, and other molecules are also transcribed via NF-κB-regulated mechanism. This disrupts the RIPK1 scaffold and consequently downregulates NF-κB activation [15–18].

Tob (Transducer of ErbB2) is one of the members of Tob/BTG (B-cell translocation gene) family protein. Tob/BTG family proteins have been discussed as anticancer molecules with many mechanisms. For example, BTG1 knockout induce the development of lymphomagenesis through the overexpression of Bcl2 [19]. BTG3 knockout keratinocytes induce the development of skin cancer by activating NF-κB in the mouse DMBA/TPA skin cancer induction model [20]. Our previous study showed that Tob-deficient mice develop tumors because Tob negatively regulates the expression of cyclin D1 [21]. Tob knockdown in human breast carcinoma cell line MCF-7 promotes tumor growth in mouse xenograft models because cyclin D1 is dysregulated [22]. In this study, we found that the constitutive activity of NF-κB negatively correlated with the expression levels of Tob in human cancer cell lines and in clinical samples referenced from the database. Since few papers have discussed the relationship between Tob and NF-κB, we further investigated the relationship between Tob and NF-κB in breast cancer. Human breast cancer cells stimulated with TNF-α showed accelerated IKK activation and increased expression of NF-κB target genes when Tob was deleted. Furthermore, we suggested that Tob interacts with RIPK1 and NEMO and becomes integrated into the TNFR complex I. These molecular mechanisms could be connected to the enhanced NF-κB activation in breast cancer in the absence of Tob.

## Results

### Tob expression is inversely correlated with NF-κB activity in breast cancer cell lines

**Fig. 1:**
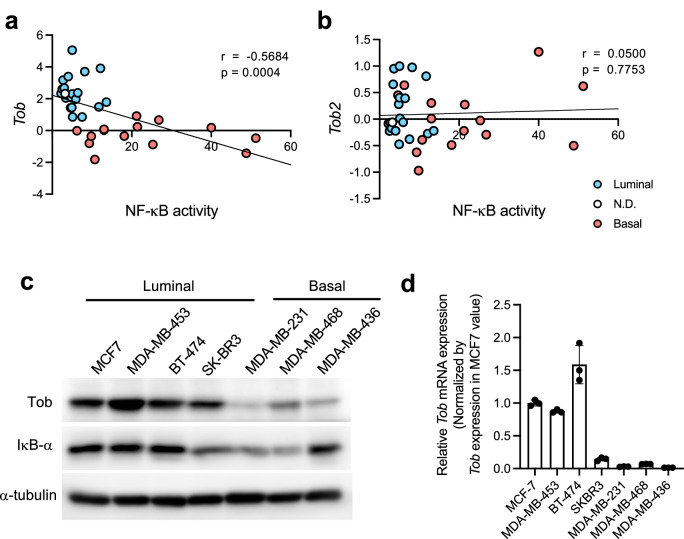
Inverse correlation of Tob expression level and NF-κB activity level in breast cancer cell lines. Correlation between *Tob* (**a**) or *Tob2* (**b**) expression and NF-κB activity level in 35 breast cancer cell lines. Red dots show basal-like subtypes, blue dots show luminal subtypes, and white dot showed not determined (N.D.). *Tob* and *Tob2* expression was from Microarray data [23]. NF-κB activity level was calculated by EMSA in previous reports [6]. *r*: Pearson’s correlation coefficient. *p*: *p*-values. *p*-values were calculated from *t*-test (*t*-value = -3.97 (**a**), = 0.288 (**b**)). **c** Western blotting of Tob in each cell lines. **d** mRNA expression level of *Tob* were measured using real-time RT-PCR. mRNA expression levels were normalized by *Gapdh*. Induction levels were calculated by dividing each *Tob* expression level by that of MCF-7. These results indicate the mean ± S.D. (*n* = 3).

To determine whether Tob expression correlates with NF-κB activity, we reexamined previously published our microarray data of 35 breast cancer cell lines [23]. Expression of Tob was low in cancers with higher NF-κB activity (Fig. 1a, b). Next, we measured the expression levels of Tob protein and mRNA using cancer cell lines that belong to different subtypes. Cell lines analyzed include MCF-7 (luminal type, ER^+^ PR^+^ ErbB2^−^), BT-474 (luminal type ER^+^ PR^+^ ErbB2^+^), MDA-MB-453, SK-BR3 (luminal type, ER^−^ PR^−^ErbB2^+^), MDA-MB-231, MDA-MB-468, and MDA-MB-436 (basal type, triple negative) (Supplementary Table 1) [6, 24, 25]. We found a trend similar to the microarray results (Fig. 1c, d). That is, cells with low Tob expression at both protein and mRNA levels, showed high NF-κB activity. We concluded that Tob expression is inversely correlated with the NF-κB activity level in breast cancer cell lines.

### Tob expression is significantly reduced in malignant breast cancer

**Fig. 2:**
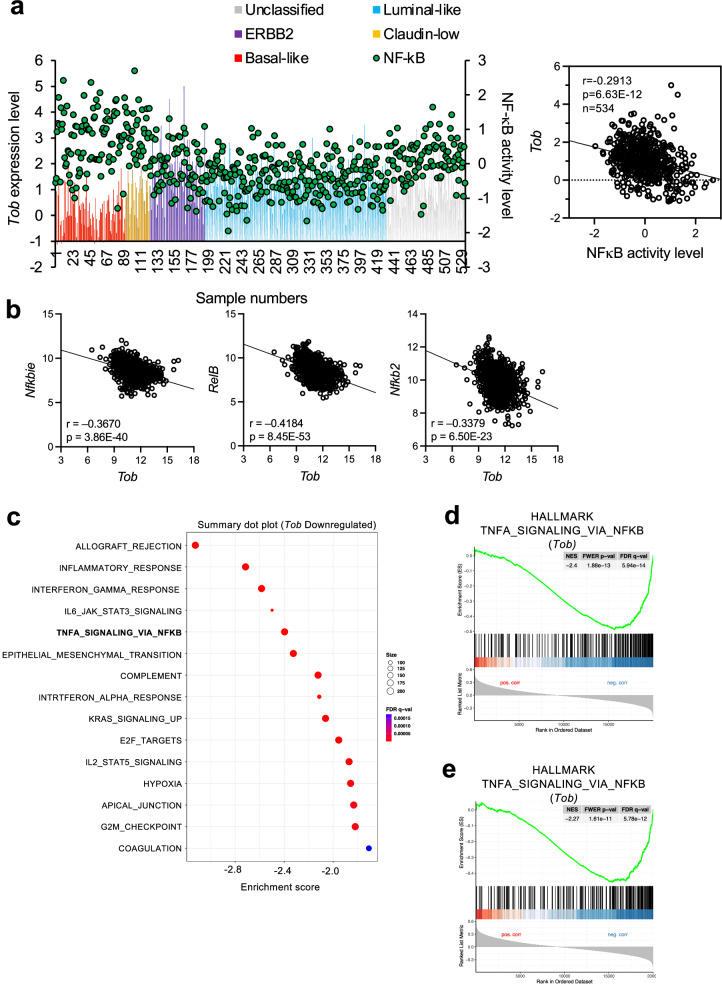
Correlations between *Tob* expression and NF-κB activity level in breast cancer patients. **a** A hierarchical clustering analysis of publicly available cDNA microarray data derived from 534 primary mammary tumor samples from TCGA described previously [26]. Left; *Tob* expression and NF-κB activity level are shown in each patient. The bar shows *Tob* expression level (left Y axis) and green dots show the NF-κB activation level (right Y axis) in each breast cancer patient. Right; Correlation between *Tob* expression and NF-κB activity level. **b** Correlation between *Tob* expression and representative genes from GSEA TIAN_TNF_SIGNALING_VIA_NFKB using TCGA data collection (TCGA_BRCA). Top 3 genes of lowest *p*-value are shown. Correlation data of all genes are shown in Supplementary Table 2. *r*: Peason’s correlation coefficient. *p*: *p*-values. *p-*values were calculated from *t*-test ((**a**) *t*-value = −7.02, (**b**) *t*-value = −13.8 (*Nfkbie*), = −16.1 (*Relb*), = (*Nfkb2*)). (**c**) GSEA summary plot using TCGA-Cell 2015 cohort [27]. Downregulated Hallmark gene sets compared to *Tob* were shown. GSEA plots of Hallmark TNFA_SIGNALING_VIA_NFKB in *Tob* expression. TCGA-Cell 2015 cohort was used in (**d**) and TCGA-PanCancerAtlas was used in (**e**). Normal *p*-value is 1.880 e^−^^14^ (**d**), 1.614 e^−12^ (**e**).

To address whether Tob expression levels correlate with breast cancer status, we scrutinized the TCGA database for 534 breast cancer patients. The link between the breast cancer subtypes and NF-κB activities was previously reported [26]. We classified patients by levels of Tob expression and NF-κB activation into the following cancer subtypes: basal-like, claudin-low, ErbB2 positive, luminal-like, and unclassified. The level of NF-κB activity was high in malignant basal-like breast cancer as reported [1, 6], whereas Tob expression in cancers of the malignant subtypes (basal-like and claudin-low) was low (Fig. 2a). We further compared the level of Tob and NF-κB activity using the TCGA-BRCA dataset and confirmed that the expression of Tob was inversely correlated with most NF-κB target genes (Fig. 2b, Supplementary Table 2). Tob2, a cousin of Tob, did not show such an apparent negative correlation with NF-κB target genes (Supplementary Fig. 1a, Supplementary Table 2). We also utilized a breast cancer patient’s cohort, Gene ENrichment Identifier (GENI) web-based tool [27, 28], and conducted gene set enrichment analysis (GSEA) (Fig. 2c–e, Supplementary Fig. 1b–f). The results revealed that the Hallmark gene set of “TNFA_SIGNALING_VIA_NFKB” was negatively correlated with Tob expression. Note that Tob2 expression showed a positive correlation with the gene set of “TNFA_SIGNALING_VIA_NFKB” in breast cancer patients (Supplementary Fig. 1e, f). These results support the assertion that Tob expression affects breast cancer malignancy.

### Tob knockdown enhanced TNF-α-induced IKK activation and NF-κB-driven transcription

**Fig. 3:**
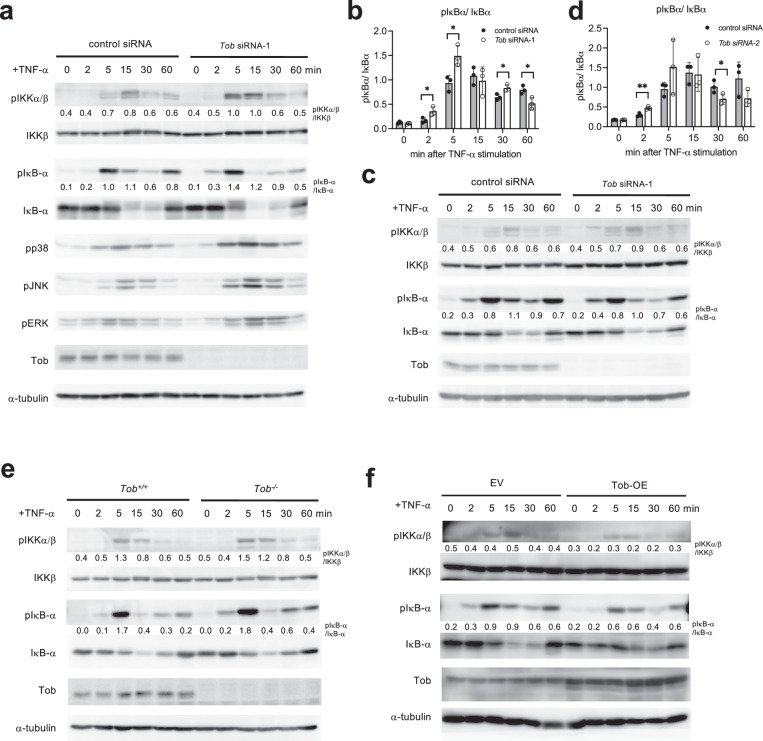
Tob acts as a negative regulator of TNF-α-induced NF-κB activation. MCF-7 cells were transiently transfected with a control, *Tob* siRNA-1 (**a**, **b**) or siRNA-2 (**c**, **d**). After 72 h, cells were treated with 10 ng/mL TNF-α. Bar graphs show the pIκB-α/ IκB-α in three independent experiments (Supplementary Fig. 2a–d). **e** Primary *Tob*^*+/+*^ or *Tob*^*-/-*^ MEFs were treated with 20 ng/mL TNF-α. **f** MCF-7 were transiently transfected with a pME18s control or *Tob* expression vector. After 24 h, cells were treated with 10 ng/mL TNF-α. Values in western blotting figures indicate the band intensity of phosphorylated proteins divided by that of total proteins. These results indicate the mean ± S.D. (*n* = 3). Statistical significance was assessed using Student’s *t-*test. *p < 0.05, **p < 0.01.

**Fig. 4:**
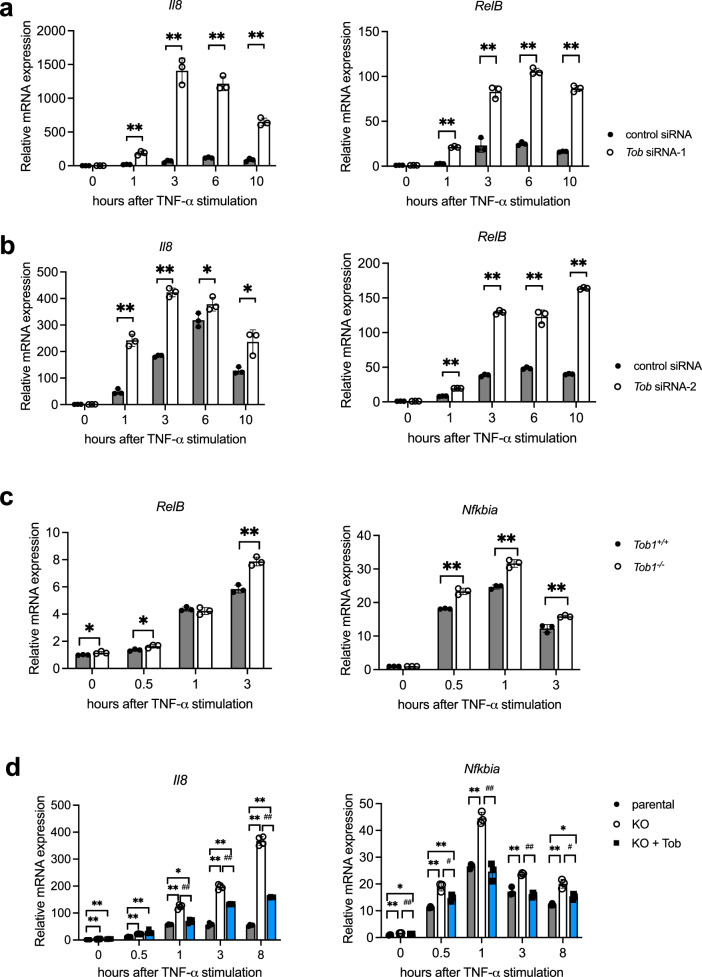
Tob downregulates transcriptions regulated by NF-κB. MCF-7 cells were transfected with a control, *Tob* siRNA-1 (**a**) or siRNA-2 (**b**). After 72 h, cells were treated with 10 ng/mL TNF-α. Expression levels of *Il8* and *RelB* were measured with real-time RT-PCR. **c** Primary *Tob*^*+/+*^ or *Tob*^*-/-*^ MEFs were treated with 20 ng/mL TNF-α. Expression levels of *RelB* and *Nfkbia* were measured with real-time RT-PCR. **d** MCF-7 parental, *Tob* KO, or *Tob* KO transfected with *Tob* expression vector was treated with 10 ng/mL TNF-α⊡ The expression level of *Il8* and *Nfkbia* was measured with real-time RT-PCR. mRNA expression levels were normalized against *Gapdh* in each sample. Fold induction was calculated by dividing expression values by that of control siRNA, *Tob*^*+/+*^ or MCF-7 parental without TNF-α stimulation. These results indicate the mean ± S.D. (*n* = 3 (**a**, **b**, **c**), *n* = 4 (**d**), technical replicates). Statistical significance was assessed using Student’s *t*-test. *p < 0.05, **p < 0.01.

To understand the molecular mechanism by which Tob functions in NF-κB signaling, we examined the effect of Tob knockdown in MCF-7 cells (Fig. 3a–d, Supplementary Fig. 2a–d). Under TNF-α treated conditions, Tob knockdown cells enhanced phosphorylation of IKK-α/β (pIKK-α/β). In the downstream pathway, Tob knockdown enhanced phosphorylation of IκB-α (pIκB-α) as early as 2–5 min after TNF-α stimulation. In line with this, phospho-p65 is upregulated upon Tob knockdown (Supplementary Fig. 2a). Moreover, Tob knockdown cells showed increased phosphorylation of p38, c-Jun N-terminal kinase (JNK), and Erk, indicative of their increased activity. Previous reports showed that JNK and p38 signaling was enhanced by TNF-α stimulation through recruitment of TAK1 to the polyubiquitin chain of RIPK1 [29, 30]. We also used different breast cancer cell lines, SK-BR3 and BT-474, and confirmed that they showed more enhancement of the pIKK-α/β and pIκB-α in Tob knockdown cells than control (Supplementary Fig. 2e, f). We confirmed enhancement of IκB-α and IKKα/β phosphorylation in mouse embryonic fibroblasts (MEFs) lacking Tob compared to controls (Fig. 3e). Tob overexpression delayed IκB-α and IKKα/β phosphorylation under TNF-α treatment in MCF-7 (Fig. 3f). On the other hand, NF-κB induced by IL-1β didn’t show the significant enhancement of IKK activation compared to TNF-α in Tob knockdown MCF-7 (Supplementary Fig. 2g–j). To determine whether Tob affects transcription of NF-κB-regulated genes, we examined mRNA levels of the NF-κB target genes. In the presence of *Tob* siRNA, TNF-α-induced expression of these genes was significantly enhanced in MCF-7 (Fig. 4a, b). In addition, expression of *RelB* and *Nfkbia*, targets of NF-κB, were enhanced in *Tob* KO MEFs (Fig. 4c). Tob overexpression in *Tob* knockout MCF-7 suppressed expression of *Il8* and *Nfkbia* mRNA (Fig. 4d). SK-BR3 and BT-474 showed *Il8* enhancement clearly, and *Nfkbia* enhancement slightly in *Tob* siRNA (Supplementary Fig. 3). These results indicate that NF-κB signaling, including IKK activation, is negatively regulated by Tob.

### Tob interacts with RIPK1 to suppress polyubiquitylation

**Fig. 5:**
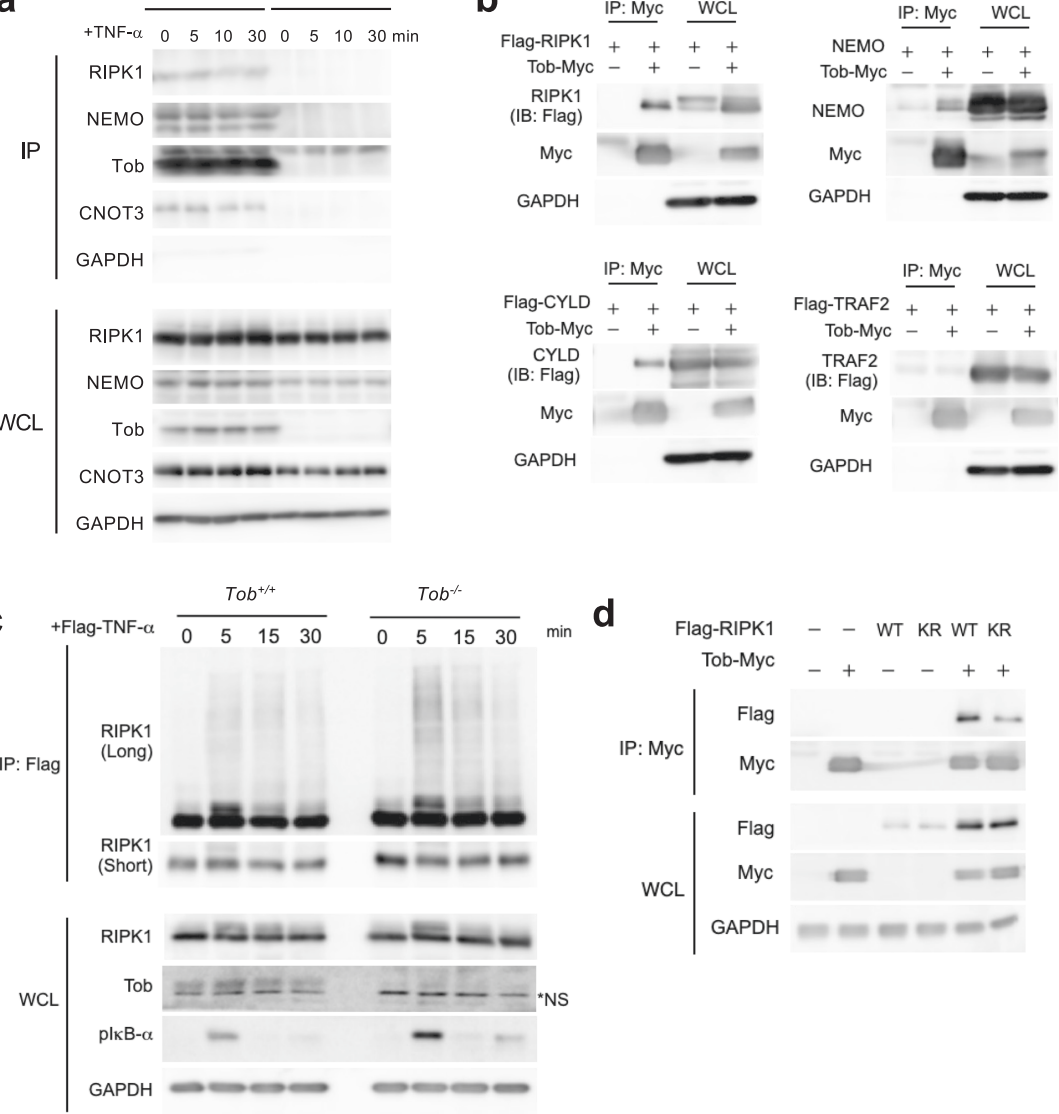
Tob interacts with TNF-receptor complex I-associated proteins. **a** MCF-7 parental or *Tob* KO were treated with 10 ng/mL TNF-α at indicated times. Cell lysates were subjected to immunoprecipitation with anti-Tob antibody. Whole cell lysates (WCL) were also prepared from the same cell lysates before performing immunoprecipitation. **b** HEK 293 T cells were transiently transfected with C-terminal Myc-tagged Tob with NEMO, N-terminal Flag-tagged TRAF2, N-terminal Flag-tagged RIPK1 or N-terminal Flag-tagged CYLD. After 24 h, cells were harvested and were subjected to immunoprecipitation with anti-Myc tag antibody. WCLs were also prepared from the same cell lysates before performing immunoprecipitation. **c** *Tob*^*+/+*^ or *Tob*^*-/-*^ MEFs were stimulated with Flag-tagged TNF-α (2 μg/mL). Cells were harvested at the indicated times and were subjected to immunoprecipitation with anti-Flag tag antibody-conjugated beads. WCLs were also prepared with the same cell lysates before performing immunoprecipitation. For the 0 min samples, 2 μg/mL of Flag-tagged TNF-α were added in post-lysis. **d** HEK 293 T cells were transiently transfected with C-terminal Myc-tagged Tob plasmids with N-terminal Flag-tagged RIPK1 parental or K377R mutant plasmids. After 24 h, cells were harvested and were subjected to immunoprecipitation as described in (**b**).

**Fig. 6:**
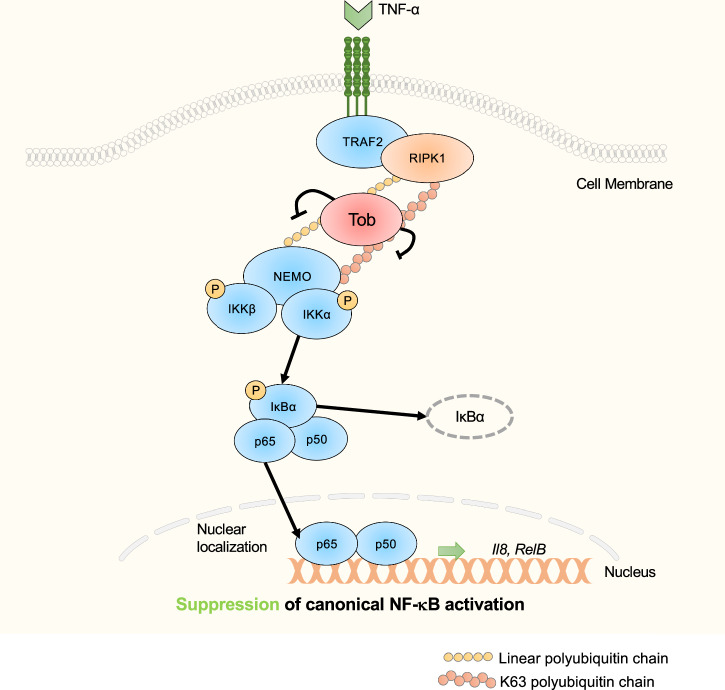
Tob interacts with polyubiquitin chains to regulate NF-kappaB signalling. The model shows that Tob associates with RIPK1 and NEMO to inhibit TNF-α signal transduction in breast cancer cells.

Because Tob knockdown enhances the TNF-α-stimulated pathway, we hypothesized that Tob functions in TNF-α dependent on canonical NF-κB signaling. The IKK complex is shown to be recruited into the TNFR complex upon TNF-α stimulation that results in the activation of IKKs. We, therefore examined an interaction of Tob with components of the TNFR and IKK complex. We found that Tob interacted with RIPK1 and NEMO in MCF-7 and SK-BR3 (Fig. 5a, Supplementary Fig. 4a, b). To examine which component of TNFR complex I binds Tob, we overexpressed NEMO, RIPK1, CYLD, or TRAF2 together with Myc-tagged Tob. Co-IP experiments showed that NEMO, RIPK1, and CYLD, but not TRAF2, are immunoprecipitated with Myc-tagged Tob (Fig. 5b, Supplementary Fig. 4c). As RIPK1 polyubiquitylation provides a platform for protein-protein interaction, we examined the relevance of RIPK1 polyubiquitylation to the interaction. After introducing Flag-TNF-α into wild-type or *Tob* KO MEFs, TNFR complex I was immunoprecipitated using an anti-Flag antibody, and coimmunoprecipitates were analyzed by gel electrophoresis and western blotting (Fig. 5c). In *Tob* KO MEFs, RIPK1 polyubiquitylation was more prominent than that in wild-type MEFs. NEMO interacts with the RIPK1 polyubiquitin chain through its ubiquitin-binding domain [8]. CYLD contains a deubiquitinating domain at its C-terminus, and, with its enzymatic activity, is able to remove M1- or Lys63-linked polyubiquitin chains from TRAF2, RIPK1, and NEMO, which suppresses NF-κB signaling [15, 16, 31–33]. Since Tob did not interact with TRAF2, we hypothesized that Tob directly associates with the RIPK1 polyubiquitin. RIPK1 polyubiquitylation, especially Lys-377 polyubiquitylation, is crucial for canonical NF-κB activation [10, 11]. To address whether Tob interacts with the RIPK1 Lys-377 polyubiquitin chain, we generated a K377R mutant of RIPK1 and performed immunoprecipitation with Tob-Myc. Tob association with RIPK1 was largely reduced by the K377R mutation (Fig. 5d). These results suggested that Tob associates with the RIPK1 polyubiquitin scaffold in the TNFR complex I and suppresses NF-κB signal transduction (Fig. 6).

## Discussion

NF-κB is essential in most of cells including immune cells, suggesting that use of therapeutic inhibitors that directly target NF-κB would cause unfavorable side effects [34]. The modulation of upstream or downstream pathways of NF-κB signaling could be an alternative approach for cancer treatment.

In the present study, we demonstrated that Tob expression is inversely correlated with NF-κB activity and with breast cancer malignancy. The negative correlation was not observed with Tob2, a close homolog (61% amino acid identity) of Tob that is implicated in TLR-induced NF-κB activation [35]. Tob-deficient mice develop various types of tumors with a relatively long latency. Tob negatively regulates the cyclin D1 gene by recruiting HDAC to the cyclin D1 promoter, which explains, at least in part, why Tob deficiency promotes spontaneous tumor development [21]. On the other hand, it is widely understood that chronic inflammation contributes to cancer development [2, 34, 36]. Canonical NF-κB activated by inflammatory cytokines leads to cell proliferation, survival, angiogenesis, and tumor invasiveness. NF-κB-induced inflammatory cytokines, such as TNF-α, IL-1β, and IL-6, promote infiltration of tumor infiltrating macrophages (TAMs) and neutrophils into the tumor microenvironment, inhibiting cytotoxic T cell functions. This may also explain malignant tumor development in the absence of Tob. Once cancer develops, tumor progression may be accelerated if Tob expression is poor.

The RIPK1 polyubiquitin scaffold is more important for resistance against immune checkpoint blockade than is its kinase domain. RIPK1 K376R mutants showed low pro-tumorigenic chemokine generation. Additionally, they showed sensitivity to TNF-induced cell death [37]. This suggests that RIPK1 polyubiquitin regulation could provide an excellent target for cancer therapy. De-ubiquitin enzyme CYLD is an important molecule to regulate the RIPK1 polyubiquitylation. As Tob and CYLD interact, we hypothesized that RIPK1 polyubiquitylation suppression by Tob would occur through the enhancement of CYLD deubiquitinating enzyme activity. However, immunoprecipitation analysis of Myc-Tob revealed that high expression of CYLD contributes to the interaction between Tob and RIPK1 in a manner independent of deubiquitinating enzyme activity. Tob does not interact with CYLD^C601S^, a deubiquitylating enzyme-inactivated mutant (Supplementary Fig. 5a). Tob appeared to be polyubiquitylated in the TNFR complex IP experiment after Flag-TNF-α stimulation (Supplementary Fig. 5b), suggesting that Tob could be deubiquitylated by CYLD. Tob also interacts with HOIP, HOIL, and SHARPIN, which are components of the linear ubiquitin chain assembly complex (LUBAC) (Supplementary Fig. 5c) [8, 36, 38]. Taken together, polyubiquitylation of Tob is likely one way to interact with TNFR complex I.

While Tob is antiproliferative, it also has multiple cellular functions. Tob interacts with poly (A)-binding protein (PABP) and CNOT7, a subunit of the CCR4-NOT complex, to regulate RNA metabolism [39–41]. On the other hand, Tob is an Erk substrate and is phosphorylated by cell cycle stimulation [42, 43]. In the immune system, Tob inhibits CD4^+^ T cell proliferation and cytokine production via association with Smad2 and Smad4 [44]. Tob KO mice have been shown to have a more aggressive phenotype, such as multiple sclerosis [45]. Now we provide the new evidence that Tob suppresses NF-κB inflammatory response. This suppression appears to be moderate, but in the long term, it is possible that Tob suppress breast cancer malignancy. Our results may provide us with opportunities to exploit Tob as an indirect therapeutic target of NF-κB in aggressive breast cancer.

### Limitations of this study and the future direction

Here, we have shown that Tob interacts with RIPK1 and NEMO and suppresses TNF-α-induced NF-κB activation by using cancer cell lines. Obviously, the use of primary cells is ideal. So far, we have conducted experiments with primary mouse fibroblasts, but it would be much better to perform experiments with mammary gland epithelial cells. In addition, we have not directly demonstrated that the interaction between Tob and TNFR complex I is related to breast cancer malignancy. It would be possible to provide this evidence using a mouse tumor model. The MMTV-PyMT spontaneous breast cancer mouse model with a mammary epithelium-specific Tob depletion should be useful [46]. While the interaction between Tob and RIPK1 is likely to be a key contributor to the excessive activation of NF-κB in the absence of Tob, it remains to be determined which part of Tob is necessary to interact with RIPK1. We have demonstrated that the RIPK1 K377 polyubiquitin chain is partially involved in the association of Tob with the TNFR complex I. To elucidate the direct interaction between Tob and the polyubiquitin chain, it is important to provide the experimental evidence that purified Tob can interact with the polyubiquitin chain. In addition, it would be crucial to show that Tob inhibits the polyubiquitylation of recombinant RIPK1 in vitro [47]. We have also raised the possibility that Tob itself is polyubiquitylated. Biochemical experiments where recombinant Tob is incubated with E1, E2, and E3 ligases would be necessary. It would be ideal if, in the future, we could provide the structure of the interaction between Tob and RIPK1 by some methods, such as X-ray crystallography [32] or AlphaFold [48]. Our preliminary AlphaFold model suggested a possible interaction between Tob and a linear di-ubiquitin chain (M1-linked). Revealing the fundamental mechanisms would be crucial to further elucidate the relationship between Tob expression and breast cancer malignancy through NF-κB activation. We have also demonstrated that Tob expression is suppressed in highly malignant basal-like and claudin-low breast cancers and shows an inverse correlation with the expression of NF-κB target genes in breast cancer cell lines and TCGA breast cancer samples. To better understand and potentially regulate the aberrant activation of NF-κB in progressive breast cancer, it would be beneficial to investigate the mechanisms underlying the downregulation of Tob expression. Further analysis of the genetic and epigenetic regulation of Tob in clinical samples will provide a more comprehensive understanding of these mechanisms.

## Materials and Methods

### Mice

C57BL/6J mice were used in this study. *Tob*^*-/-*^ mice were generated by Yoshida et al. [49], who describe validation and genotyping methods. The mice were used for preparation of embryonic fibroblasts.

### Plasmids

Human cDNA encoding Tob in vector pME18s was generated as previously described [42]. Human Tob and TRAF2 cDNAs were generated by PCR and cloned in plasmid pCMV-HA (635690, Clontech, Mountain View, CA, USA) or pCMV-Flag (635688, Clontech) (Primers for human Tob, forward: GATCGAATTCGGATGCAGCTTGAAATCCAAGTAGCAC, reverse: GATCCTCGAGATTAGTTAGCCATAACAGGCTGGAATTGC. Primers for human TRAF2, forward: ATGCGTCGACCATGGCTGCAGCTAGCGTGAC, reverse: GCATGCGGCCGCTTAGAGCCCTGTCAGGTCCAC). pRK5-human NEMO and pRK5-Flag-human CYLD were as described in [47]. pRK5-Flag-human RIPK1 was kindly obtained from Hiroyasu Nakano (Toho University). pcDNA3.1-Flag-His6-human CYLD (WT), and pcDNA3.1-Flag-His6-human CYLD (C601S) were kindly obtained from Fuminori Tokunaga (Osaka Metropolitan University). pRK5-Flag-human RIPK1 K377R mutants were generated from pRK5-Flag-human RIPK1 using a KOD-Plus mutagenesis kit (SMK-101, TOYOBO, Osaka, Japan) (Primers, forward: GCAGAGTAGACTCCAAGACGAAG, reverse: AGGCTGGGCTCATTCTCTTC). pCMV3-Flag8-SHARPIN (#50014), pCMV3-Flag8-HOIP (#50015), and pCMV3-Flag8-HOIL-1L (#50016) were purchased from Addgene (Cambridge, MA, USA).

### Antibodies

The following antibodies were used; anti-p-IκBα (#9246, Cell Signaling Technology (CST), Danvers, MA, USA), anti-IκBα (#9242, CST), anti-p-IKKα/β (#2697, CST), anti-IKKβ (#8943, CST), anti-NEMO (#2685, CST), anti-RIPK1 (#3493, CST), anti-p65 (#8242, CST), anti-phospho-p65 (#3033, CST), anti-p-p38 MAPK (#8690, CST), anti-p-JNK/SAPK (#4671, CST), anti-p-p44/42 MAPK (#9101, CST), and anti-GAPDH (#2118, CST), anti-α-tubulin (T9026, Merck, Darmstadt, Germany), and anti-M2-Flag tag (F1804-1MG, Merck), anti-Flag tag (PM020, MBL, Tokyo, Japan), anti-Myc tag (rabbit IgG, 562-5, MBL), and anti-Myc tag (mouse IgG, M192-3, MBL), anti-CNOT3 (cl.54, Biomatrix, Chiba, Japan), anti-Tob polyclonal for WB (#116163, NovoPro, Shanghai, China), anti-Tob monoclonal antibody for IP and WB was generated our laboratory as described in our previous report [50].

### Recombinant proteins

Following recombinant proteins were used for cell stimulation; human TNF-α (210-TA-020, R and D systems, Minneapolis, MN, USA), and mouse TNF-α (410-MT-010, R and D systems), human-IL-1β (200-01B, Peprotech, Rocky Hill, NJ, USA), mouse Flag-tagged TNF-α (ALX-522-009, Enzo Life Sciences, Farmingdale, NY, USA).

### Cell culture and transfection

HEK293T, MDA-MB-436, MDA-MB-468, MDA-MB-231, SK-BR3, BT-474, MDA-MB-453, MCF-7, and MEF cells were maintained in Dulbecco’s modified Eagle’s medium (DMEM) supplemented with 10% heat-inactivated fetal bovine serum (Merck) and penicillin-streptomycin solution (15140-122, Thermo Fisher Scientific, Waltham, MA, USA). Plasmid transfection into MCF-7 or HEK293T cells was performed with TransIT-LT1 Transfection reagent (V2306, Mirus Bio LCC., Madison, WI, USA) according to the manufacturer’s method using Opti-MEM™ I Reduced Serum Medium (31985062, Thermo Fisher Scientific). siRNA transfection into MCF-7, SK-BR3 or BT-474 cells was performed using Lipofectamine RNAiMax transfection reagent (13778-150, Thermo Fisher Scientific) according to the manufacturer’s method using Opti-MEM™ I Reduced Serum Medium (31985062, Thermo Fisher Scientific). The following target sequences were used: *Tob* siRNA-1 sense/anti-sense, 5’-CAUUUUGGUAGAGCCGAACTT-3’/5’-GUUCGGCUCUACCAAAAUGTT-3’; *Tob* siRNA-2 sense/anti-sense, 5’-CAAGGUUGCACGUACUUCUCC-3’/5’-AGAAGUACGUGCAACCUUGUU-3’. Control sense/anti-sense, 5’-UUCUCCGAACGUGUCACGUTT-3’/5’-ACGUGACACGUUCGGAGAATT-3’ from Invitrogen (Carlsbad, CA, USA).

### Generation of Tob knockout cells

Guide RNA oligos were selected by Guide Design Resources (https://zlab.bio/guide-design-resources). Forward oligo 5’-caccgCTAAACCCCGATCCTTTGTA-3’ and reverse oligo 5’-aaacTACCCAAAGGATCGGGGTTTAGc-3’ were cloned into pSpCas9(BB)-2A-puro [51]. Cells transfected with guide RNA expression plasmids were selected with 2 μg/mL puromycin 48 h after transfection. Single cells were re-seeded and cultured with DMEM supplemented with 10% heat-inactivated fetal bovine serum as described above. Tob knockout cell lines were verified by western blot and DNA sequencing. To restore Tob expression in Tob KO cells, Tob KO MCF-7 cells were infected with retroviral plasmid pMX-puro carrying human Tob cDNA (pMX-Tob) [42]. In detail, the Platinum-A retroviral packaging cells (2 × 10^6^) (Cell Biolabs, CA, USA) were seeded on 60 mm dish and cultured in DMEM with 10% heat-inactivated fetal bovine serum for 24 h. After the culture medium had been replaced with 5 mL of fresh medium, the transfection was performed with 2 μg of pMXs human-Tob or empty vector plasmid. After 48 h incubation, the supernatant was passed through a 0.45 μm filter and applied to MCF-7 *Tob* KO cells with 5 μg/mL polybrene (107689, Merck) and added 8 mL culture medium 5 h later. On day 2 following infection, the transduced cells were selected with 5 μg/mL puromycin for 21 days, and then cells were cultured with a culture medium until the experiment.

### Immunoprecipitation

Cells were washed with cold PBS and lysed in TNEN buffer (20 mM Tris-HCl [pH7.5], 150 mM NaCl, 1 mM EDTA, 1% NP-40) with PhosSTOP Phosphatase Inhibitor Cocktail (4906837001, Roche, Basel, Switzerland), and Protease Inhibitor Cocktail (EDTA-free) (03969-21, Nacalai tesque, Kyoto, Japan), followed by centrifugation at 16,500 *g* for 10 min at 4 °C to remove insoluble material. For immunoprecipitation, cell lysates were incubated with 1-2 μg antibodies and protein G-Sepharose (17-0618-02, GE Healthcare, Chicago, IL, USA) or Dynabeads (10004D, Thermo Fisher Scientific, Waltham, MA, USA) at 4 °C. Immunoprecipitants were washed five times with TNEN buffer or 0.05% SDS-contained TNEN buffer. After boiling in 1× SDS sample buffer, samples were subjected to immunoblotting. For TNFR complex I purification, MEFs were seeded at 1.25 × 10^6^ cells per 150 mm dish and treated with 2 μg/mL of Flag-tagged TNF-α. Following the removal of the media, the plates were washed with cold phosphate-buffered saline (PBS) and frozen at −80 °C. The plates were thawed on ice. Cells were lysed with lysis buffer (30 mM Tris-HCl [pH7.5], 120 mM NaCl, 2 mM EDTA, 2 mM KCl, 10% glycerol, 1% Triton X-100, and Phosphatase Inhibitor and Protease Inhibitor Cocktail [EDTA-free] tablets, as indicated), followed by centrifugation at 16,500 *g* for 10 min at 4 °C to remove the insoluble fraction. Cell supernatants were incubated on a rotating incubator overnight at 4 °C with 20 μL of α-Flag M2 beads (A2220, Merck). For the time 0 samples, 2 μg/mL of Flag-tagged TNF-α was added to post-lysis samples. Beads were washed with lysis buffer four times, and samples were eluted by boiling in 60 μL 1× SDS sample buffer [52].

### Immunoblotting

For immunoblotting, immunoprecipitants or cell lysates were separated by SDS-polyacrylamide gel electrophoresis (PAGE) and transferred to poly vinylidene fluoride membranes (Immobilon P; Merck). These membranes were incubated with primary antibodies, and immunoreactive proteins were visualized with horseradish peroxidase-conjugated secondary antibodies (GE Healthcare, Chicago, IL, USA) and an ECL Western Blotting Detection System (GE Healthcare). Band intensity was quantified by Image J (National Institutes of Health).

### Quantitative real-time reverse transcriptase PCR

Total RNA was isolated from cells using ISOGEN II (311-07361, Nippon Gene, Tokyo, Japan) according to the manufacturer’s method. cDNA was synthesized from 1 μg of RNA with SuperScript III Reverse Transcriptase (18080-085, Thermo Fisher Scientific) according to the manufacturer’s method. Quantitative real-time PCR analysis was performed on a ViiA^TM^7 real-time PCR system (Thermo Fisher Scientific) using SYBR Green reagents (RR820, TaKaRa, Shiga, Japan). The level of GAPDH expression was used to normalize expression data. The following primers were used: human *Tob* sense/anti-sense, 5’-TCTGTATGGGCTTGGCTTG -3’/5’- GTTGCTGCTGTGGTGGTG -3’ human *Il8* sense/anti-sense, 5’-ATGACTTCCAAGCTGGCCGT-3’/5’-TTACATAATTTCTGTGTTGGC-3’; human *RelB* sense/anti-sense, 5’-ATTTGCCGAATTAACAAGGA-3’/5’-CCTGCTGAACACCACTGATA-3’; mouse *RelB* sense/anti-sense, 5’- GTGTTCTTGGACCACTTCCT -3’/5’- GAAGCAGGGAAGAAATCAGA -3’; human *Nfkbia* sense/anti-sense, 5’- TTCAGATGCTGCCAGAGAGT -3’/5’- CCTCCAAACACACAGTCATC -3’;mouse *Nfkbia* sense/anti-sense, 5’-TGACTTTGGGTGCTGATGT-3’/5’-ATTTCAACAAGAGCGAAACC-3’; human *Gapdh* sense/anti-sense, 5’-GAAGGTGAAGGTCGGAGTCA-3’/5’-TTGATGGCAACAATATCCACTT-3’; mouse *Gapdh* sense/anti-sense; 5’-CTGCACCACCAACTGCTTAG-3’/5’-GTCTTCTGGGTGGCAGTGAT-3’.

### Data analysis

Microarray analysis of 35 breast cancer cell lines was performed previously [23]. NF-κB activation levels were calculated by EMSA results performed in previous report [6].

### Clinical analysis

A hierarchical clustering analysis of publicly available cDNA microarray data derived from 534 primary mammary tumor samples from The Cancer Genome Atlas (tcga-data.nci.nih.gov/) was described previously [26]. Evaluations of NF-κB activation levels were also described in a previous report [26]. GSEA in breast cancer patients was performed with GENI (https://yoavshaul-lab.shinyapps.io/gsea-geni/) [28] using two Breast Invasive Carcinoma cohorts (TCGA, Cell 2015 [27] and PanCancer Atlas) with Hallmark gene sets.

### Statistical analysis

Experimental results were expressed as the mean ± SD. The data were subjected to an unpaired Student’s *t*-test (two-tailed) to determine the statistical significance. Pearson’s correlation coefficient and *p*-values were calculated by Microsoft excel. Graphs were drawn by GraphPad Prism of Microsoft excel.

## Supplementary information

Supplemental material

## Data Availability

The Microarray data were firstly generated by Ito et al. [23] and were available in the DNA Data Bank of Japan (DDBJ) via the Center for Information Biology gene EXpression (CIBEX) database (accession number; CBX20).
