# Supplementary material for: Tob negatively regulates NF-κB activation in breast cancer through its association with the TNF receptor complex
Source: Cancer Gene Ther. 2025 Apr 1;32(5):573–83. doi: 10.1038/s41417-025-00897-6 (PMC12086088; doi:10.1038/s41417-025-00897-6)
Supplement: Supplementary file 1 — Supplemental material [file 41417_2025_897_MOESM1_ESM.pdf]

Tob negatively regulates NF-κB activation in breast cancer through its association with the TNF receptor complex.

Miho Tokumasu*[1,2], Atsuko Sato*[1], Taku Ito-Kureha[1,3], Mizuki Yamamoto[4], Nao Ohmine[1], Kentaro Semba[5], Jun-ichiro Inoue[6], Tadashi Yamamoto[1].

[1] Cell Signal Unit, Okinawa Institute of Science and Technology Graduate University, Okinawa, Japan.
[2] Department of Immunology, Okayama University Graduate School of Medicine, Dentistry, and Pharmaceutical Sciences, Okayama, Japan.
[3] Department of Immunology, Graduate School of Medicine and Faculty of Medicine, The University of Tokyo, Tokyo, Japan.
[4] Research Center for Asian Infectious Diseases, The Institute of Medical Science, The University of Tokyo, Tokyo, Japan.
[5] Department of Life Science and Medical Bioscience, Waseda University, Tokyo, Japan.
[6] The University of Tokyo Pandemic Preparedness, Infection and Advanced Research Center (UTOPIA),Tokyo, Japan.

* Miho Tokumasu and Atsuko Sato is equally contributed.

Correspondence:
Tadashi Yamamoto, PhD
Okinawa Institute of Science and Technology Graduate University.
1919-1 Tancha, Onna-son, Kunigami-gun Okinawa, Japan 904-0495
Tel: +81-98-966-8732, Fax: +81-98-966-1084, E-mail. tadashi.yamamoto@oist.jp
Miho Tokumasu, PhD
Department of Immunology, Okayama University Graduate School of Medicine, Dentistry, and Pharmaceutical Sciences.
2-5-1 Shikata-cho, Kita-ku, Okayama, Japan 700-8558
Tel: +81-86-235-7192, Fax: +81-86-235-7193, E-mail. pnc89cju@s.okayama-u.ac.jp

Competing Interests
We have nothing to disclose.

Supplementary Figure 1

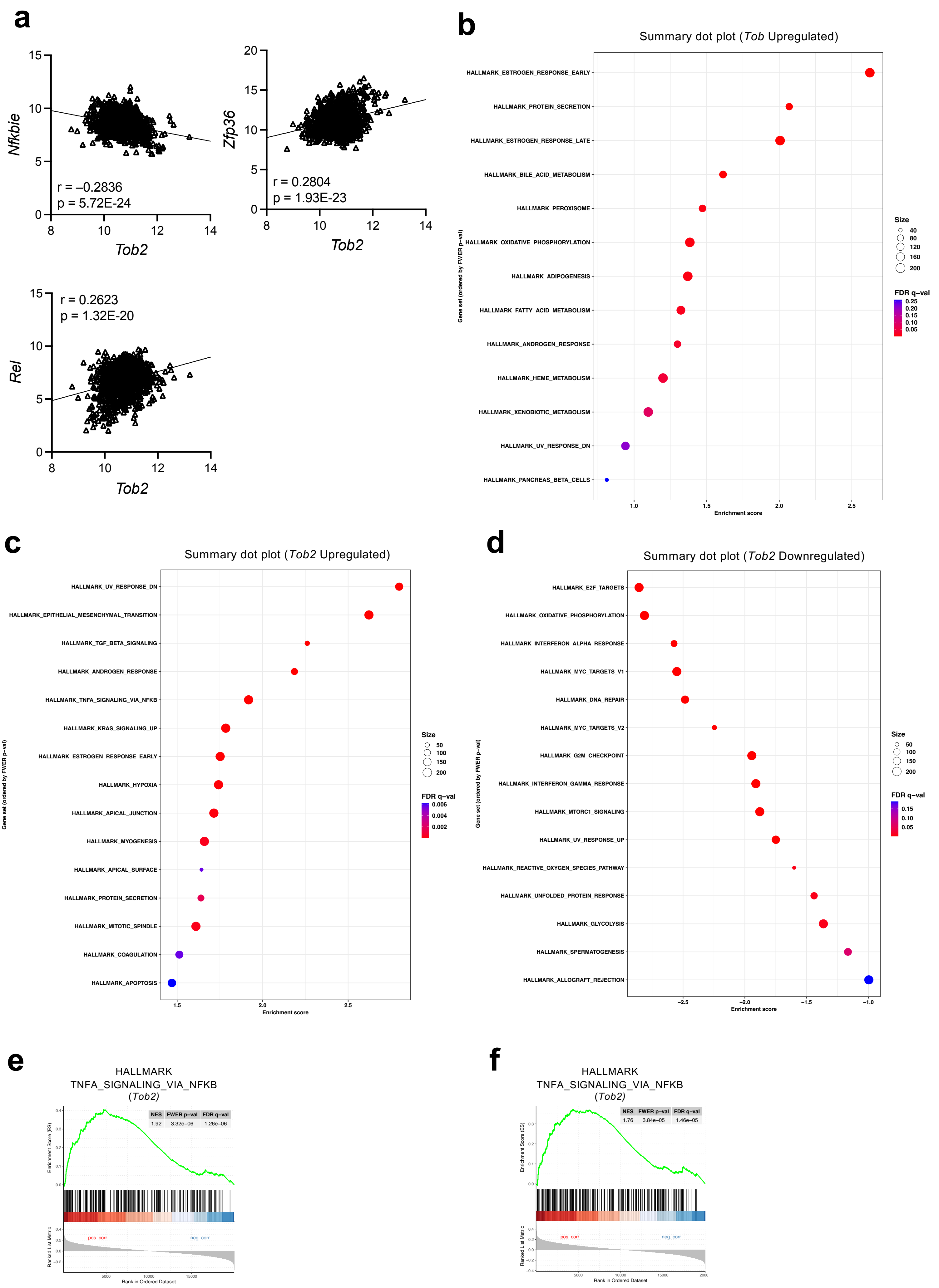

Supplementary Figure 1. (a) Correlation between *Tob2* expression and representative genes from GSEA TIAN_TNF_SIGNALING_VIA_NFKB using TCGA data collection (TCGA_BRCA). Top 3 genes with the lowest *p*-value are shown. Correlation data of all genes are shown in Supplementary Table 2. *r*: Peason's correlation coefficient. *p*: *p*-values. *p*-values were calculated from *t*-test (*t*-value = -10.3 (*Nfkbie*), = 0.280 (*Zfp36*), = 9.48 (*Rel*)). (b-d) GSEA using TCGA-Cell 2015 cohort using GENI. (b) Upregulated Hallmark gene sets compared to *Tob* are shown. (c) Upregulated Hallmark gene sets compared to *Tob2* are shown. (d) Downregulated Hallmark gene sets compared to *Tob2* are shown. (e, f) GSEA plots of Hallmark TNFA_SIGNALING_VIA_NFKB in *Tob2* expression. TCGA-Cell 2015 cohort is used in (e) and TCGA-PanCancerAtlas is used in (f). Normal *p*-value is 7.964 e^{-07} (e), 1.230 e^{-05} (f).

Supplementary Figure 2

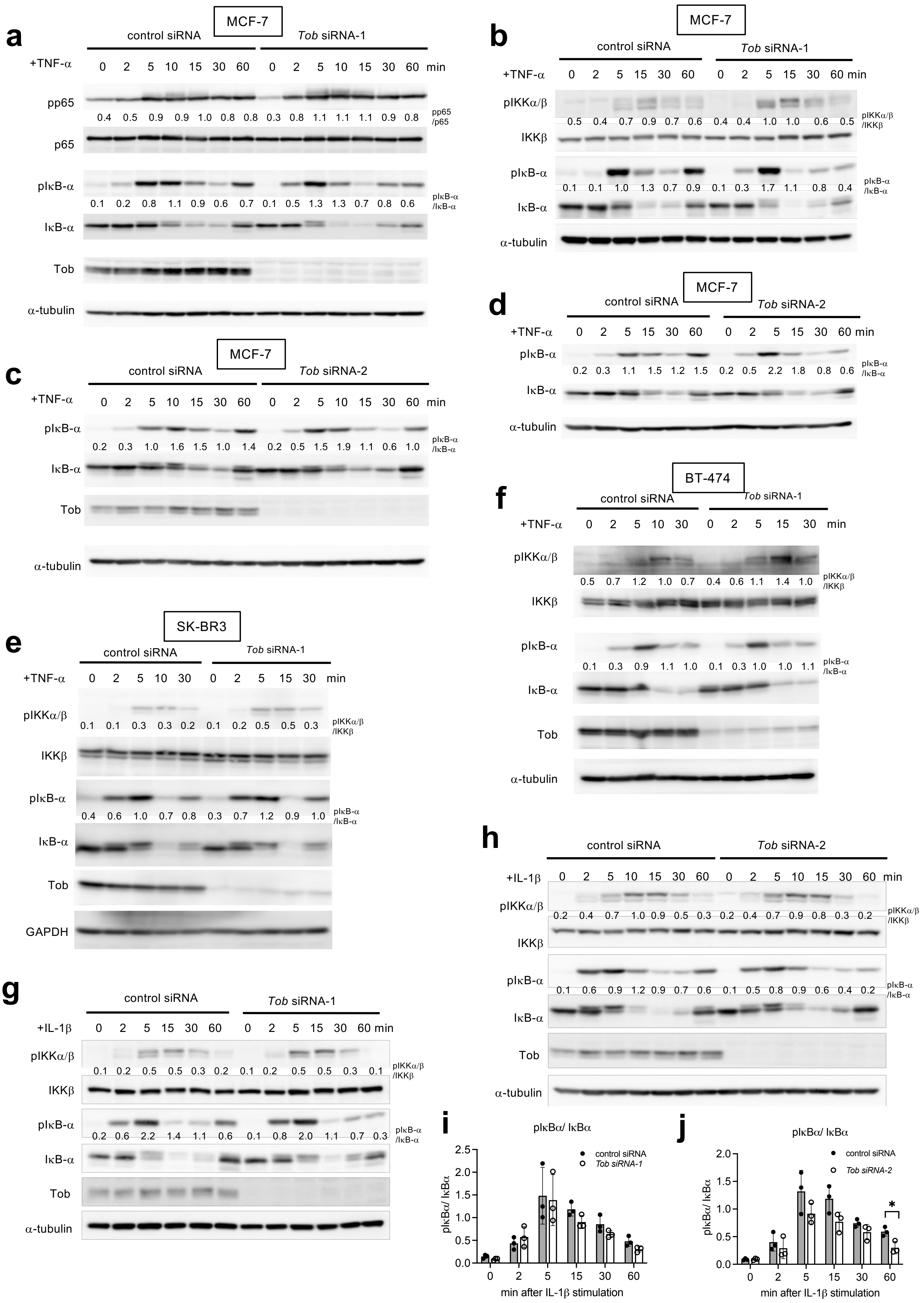

Supplementary Figure 2: Tob knockdown enhances NF-κB activation by TNF-α rather than IL-1β. (a-d) MCF-7 cells were transiently transfected with a control, *Tob* siRNA-1 or -2. After 72 h, cells were treated with 10 ng/mL TNF-α, related to Figure 3 a-d. (e, f) SK-BR3 cells (e) or BT-474 cells (f) were transiently transfected with a control, *Tob* siRNA-1. After 72 h, cells were treated with 20 ng/mL TNF-α. (g-j) MCF-7 cells were transiently transfected with a control, *Tob* siRNA-1 (g, i) or siRNA-2 (h, j). After 72 h, cells were treated with 10 ng/mL IL-1β. Bar graphs show the pIκB-α/IκB-α in three independent experiments. Values in western blotting figures indicate the the band intensity of phosphorylated proteins divided by that of total proteins. These results indicate the mean ± S.D. (n=3). Statistical significance was assessed using Student's *t*-test. *$p < 0.05$.

Supplementary Figure 3

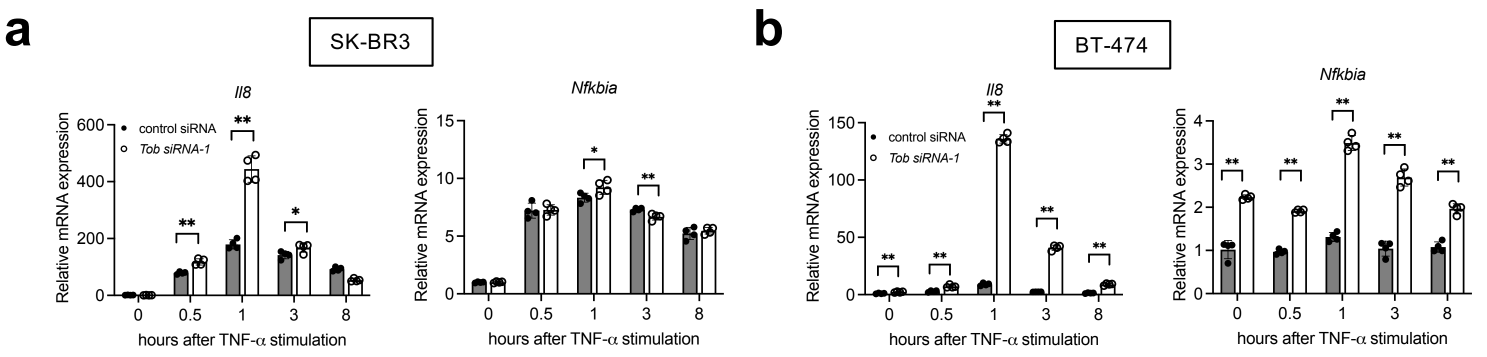

Supplementary Figure 3: Tob downregulates transcriptions regulated by NF-κB. (a, b) SK-BR3 cells (a) or BT-474 cells (b) were transfected with a control or *Tob* siRNA-1. After 72 h, cells were treated with 20 ng/mL TNF-α. Expression levels of *Il8* and *Nfkbia* were measured with real-time RT-PCR. mRNA expression levels were normalized against *Gapdh* in each sample. Fold induction was calculated by dividing expression values by that of control siRNA without TNF-α stimulation. These results indicate the mean ± S.D. (n=4, technical replicates). Statistical significance was assessed using Student's *t*-test. *$p < 0.05$, **$p < 0.01$.

Supplementary Figure 4

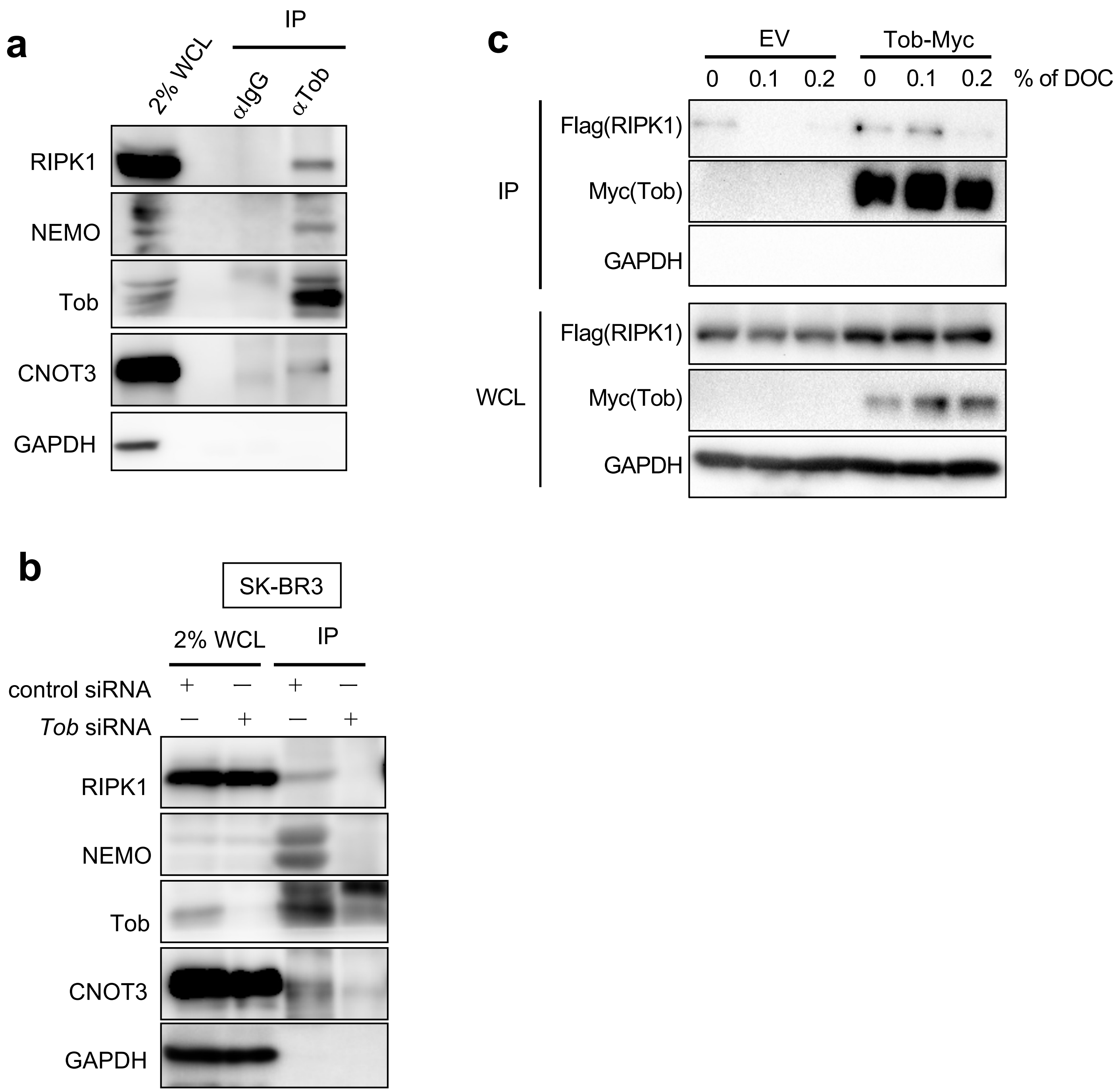

Supplementary Figure 4 : Tob interact with RIPK1 and NEMO. (a) Immunoprecipitation by anti-Tob antibody or IgG control with MCF7 lysate. (b) Immunoprecipitation by anti-Tob antibody with SK-BR3 cell lysate transfected with control siRNA or *Tob* siRNA-1. (c) HEK 293 T cells were transiently transfected with C-terminal Myc-tagged Tob with N-terminal Flag-tagged RIPK1. After 24h, cells were harvested with TNEN lysis buffer with different concentration of deoxycholate (DOC). Samples were subjected to immunoprecipitation with anti-Myc tag antibody.

Supplementary Figure 5

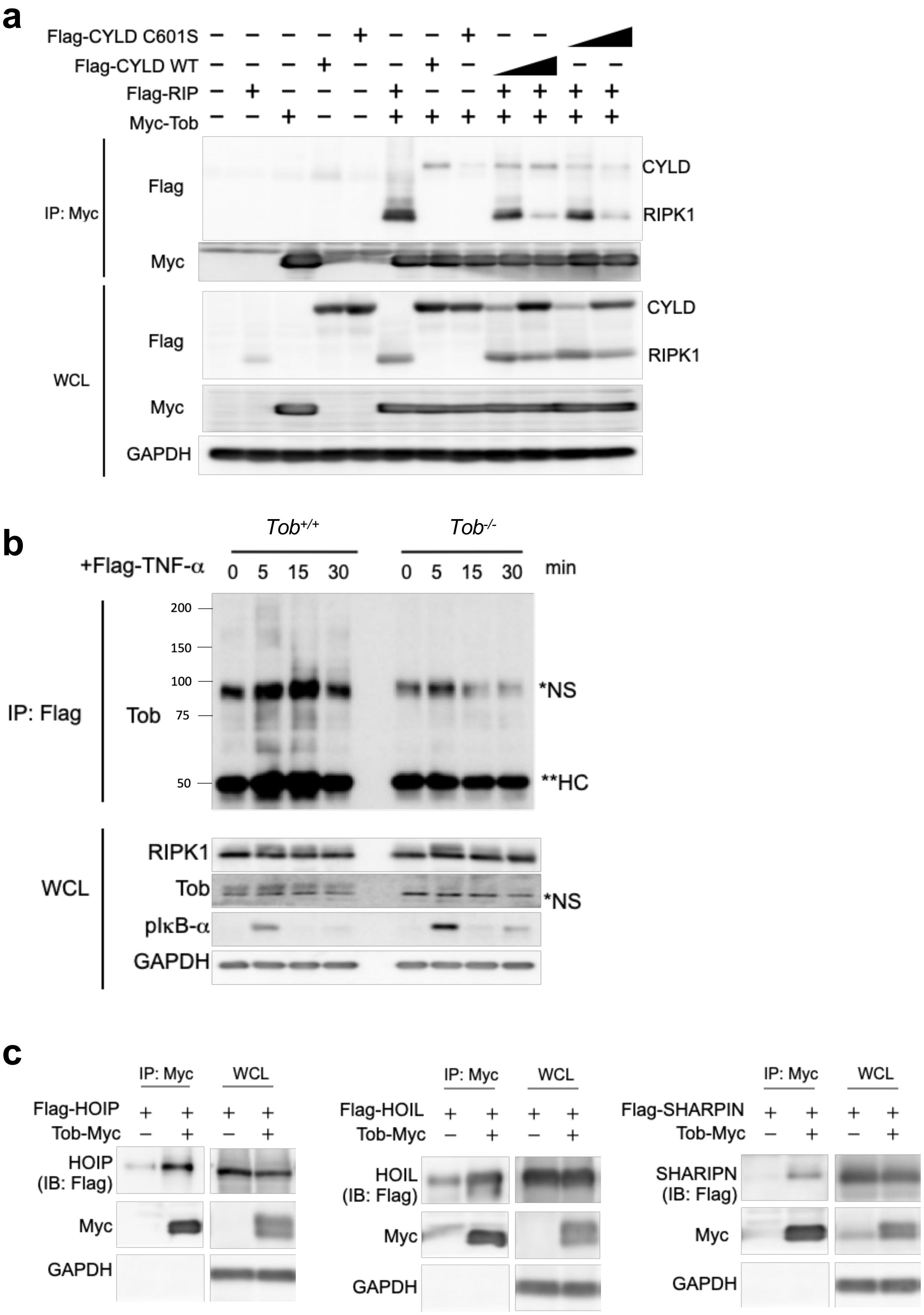

Supplementary Figure 5: High expression of CYLD prevents Tob and RIPK1 interaction independently of its de-ubiquitylation activity. (a) HEK293T cells were transiently transfected with C-terminal Myc-tagged Tob (1 μg), N-terminal Flag-tagged RIPK1 (0.2 μg), and N-terminal Flag-tagged CYLD WT (0.3 μg or 3 μg) or $CYLD^{C601S}$ mutant (0.3 μg or 3 μg). After 24 h, cells were harvested and subjected to immunoprecipitation with an anti-Myc tag antibody and protein G-conjugated beads. WCLs were also prepared from the same cell lysates before immunoprecipitation. (b) $Tob^{+/+}$ or $Tob^{-/-}$ MEFs were stimulated with Flag-tagged TNF-α (2 mg/mL). Cells were harvested at the indicated times and were subjected to immunoprecipitation with anti-Flag tag antibody-conjugated beads (refer to Figure 5c). IP samples were subjected to western blotting with anti-Tob antibody. (c) HEK293T cells were transiently transfected with C-terminal Myc-tagged pME18s-Tob and N-terminal Flag-tagged HOIP, HOIL, or SHARIPN. After 24 h, cells were harvested and subjected to immunoprecipitation with an anti-Myc tag antibody and protein G-conjugated beads. WCLs were also prepared from the same cell lysates before immunoprecipitation.

Supplementary Table 1

Cell line	subtype	ER	PR	ErbB2
CRL1500	luminal	+	+/—	—
UACC-812	luminal	+	+/—	+
HCC1419	luminal	—	—	+
T47D	luminal	+	+	—
YMB-1-E	ND			
MCF-7	luminal	+	+	—
MDA-MB-453	luminal	—	—	+
MDA-MB-361	luminal	+	+	+
HCC2157	basal	—	—	—
MDA-MB-175VII	luminal	+	—	—
HCC1500	luminal	+	+	—
HCC2218	luminal	—	—	+
UACC-893	luminal	—	—	+
MDA-MB-415	luminal	+	+/—	—
BT-483	luminal	+	+/—	—
HCC70	basal	—	—	—
BT-474	luminal	+	+	+
MDA-MB-134VI	luminal	+	—	—
ZR-75-30	luminal	+	—	+
DU4475	basal	—	—	—
MDA-MB-157	basal	—	—	—
Hs 578T	basal	—	—	—
CAMA-1	luminal	+	+/—	—
HCC202	luminal	—	—	+
BT-20	basal	—	—	—
HCC1954	basal	—	—	+
SK-BR-3	luminal	—	—	+
MDA-MB-231	basal	—	—	—
HCC1937	basal	—	—	—
MDA-MB-468	basal	—	—	—
BT-549	basal	—	—	—
HCC38	basal	—	—	—
HCC1143	basal	—	—	—
MDA-MB-436	basal	—	—	—
HCC1395	basal	—	—	—

Supplementary Table 1: Subtypes of breast cancer cell lines, presented in references 6, 24, 25.

Supplementary Table 2

Gene Symbol	Entrez Gene ID	TOB1		TOB2	
		correlation (r)	p value	correlation (r)	p value
RELB	5971	-0.4184	8.45E-53	-0.1396	0.000001001
NFKBIE	4794	-0.367	3.86E-40	-0.2836	5.72E-24
NFKB2	4791	-0.3379	6.50E-34	-0.1407	8.29E-07
TNIP1	10318	-0.3309	1.63E-32	0.1069	0.0001859
CXCL1	2919	-0.3172	7.20E-30	0.02792	0.3302
CCL20	6364	-0.2771	6.52E-23	-0.1148	0.00005905
SDC4	6385	0.2734	2.55E-22	-0.05254	0.0668
CXCL3	2921	-0.2593	3.70E-20	0.08411	0.003307
CD83	9308	-0.241	1.47E-17	0.01743	0.5434
GFPT2	9945	-0.2382	3.62E-17	0.1316	0.000004057
TRAF1	7185	-0.2308	3.48E-16	-0.02989	0.2973
IL8	3576	-0.2154	2.96E-14	0.08135	0.004497
REL	5966	0.2096	1.48E-13	0.2623	1.32E-20
TNFAIP2	7127	-0.2034	7.66E-13	0.06684	0.01966
PTGS2	5743	-0.1595	2.18E-08	0.2203	7.44E-15
TNFAIP3	7128	-0.1544	6.16E-08	0.1506	1.28E-07
IRF1	3659	-0.1539	6.72E-08	-0.05156	0.07207
CXCL2	2920	-0.1482	2.05E-07	0.1492	1.70E-07
TNFRSF9	3604	-0.1369	0.00000161	-0.06676	0.0198
IL6	3569	-0.1244	0.00001334	0.1736	1.08E-09
PLK2	10769	0.1086	0.0001464	0.1227	0.00001767
FST	10468	-0.08853	0.001985	0.09241	0.001244
NFKB1	4790	-0.08431	0.003234	0.2505	6.94E-19
NFKBIA	4792	-0.06722	0.01896	0.02084	0.4673
GCH1	2643	0.06611	0.02104	-0.133	0.000003212
CTGF	1490	-0.05939	0.03822	0.1334	0.000002981
ZFP36	7538	0.04923	0.08588	0.2804	1.93E-23
IL7R	3575	-0.01387	0.6287	0.1452	3.61E-07

Supplementary Table 2: Gene set from GESA TIAN_TNF_SIGNALING_VIA_NFKB. The genes shown in red are the three with the lowest *p*-values and are shown in Figure 2b and Supplementary Figure 1a.
